# Supplementary material for: The causal role of circulating inflammatory markers in osteoporosis: a bidirectional Mendelian randomized study
Source: Front Immunol. 2024 Jul 18;15:1412298. doi: 10.3389/fimmu.2024.1412298 (PMC11291241; doi:10.3389/fimmu.2024.1412298)
Supplement: Supplementary file 1 [file DataSheet_1.zip › supplementary material/CIM-OSTEOPOROSIS /GCST90274818.csv_forest.pdf]

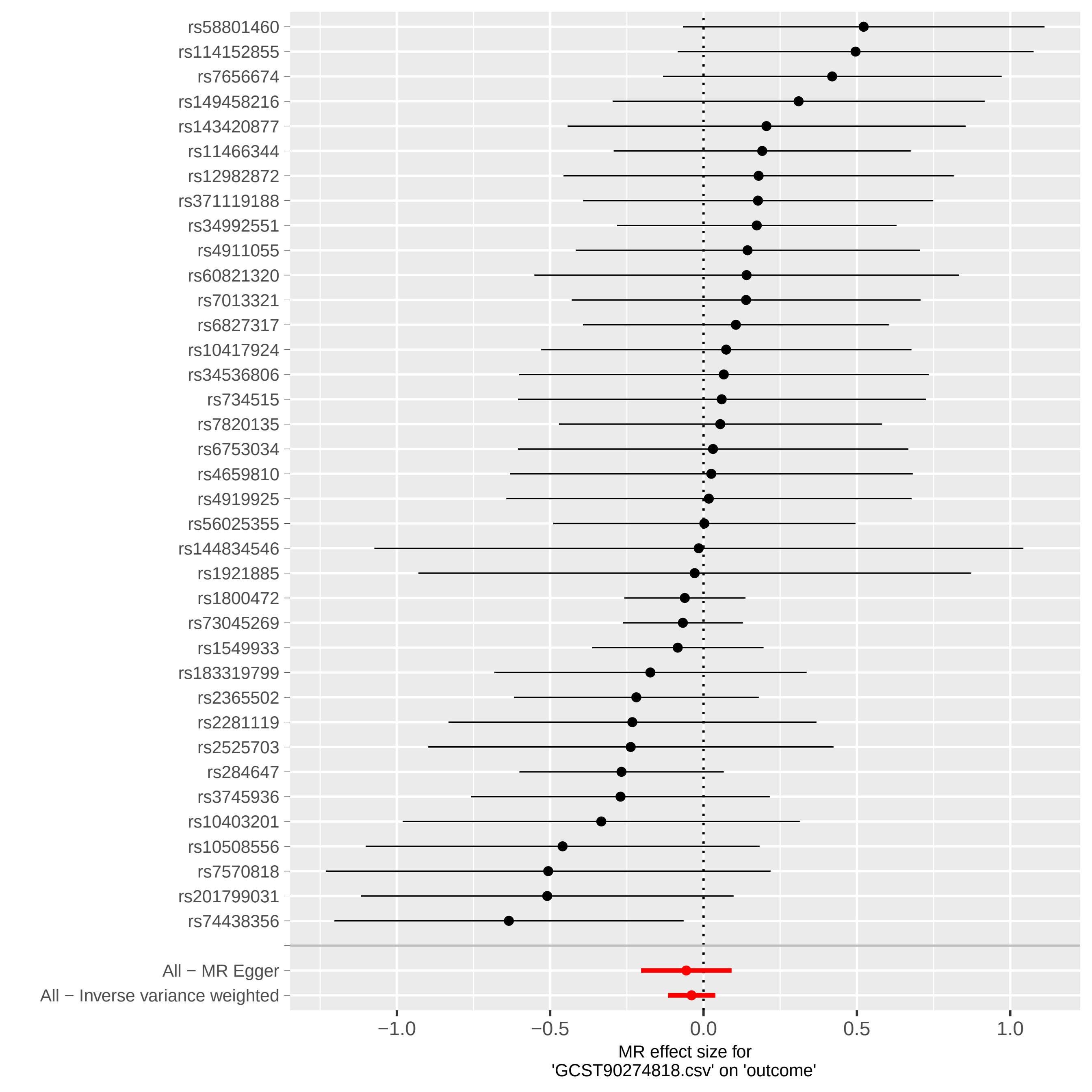
rs58801460
rs114152855
rs7656674
rs149458216
rs143420877
rs11466344
rs12982872
rs371119188
rs34992551
rs4911055
rs60821320
rs7013321
rs6827317
rs10417924
rs34536806
rs734515
rs7820135
rs6753034
rs4659810
rs4919925
rs56025355
rs144834546
rs1921885
rs1800472
rs73045269
rs1549933
rs183319799
rs2365502
rs2281119
rs2525703
rs284647
rs3745936
rs10403201
rs10508556
rs7570818
rs201799031
rs74438356
All − MR Egger
All − Inverse variance weighted
−1.0
−0.5
0.0
0.5
1.0
MR effect size for
'GCST90274818.csv' on 'outcome'
